# Sensing Method for Wet Spraying Process of Tunnel Wall Based on the Laser LiDAR in Complex Environment

**DOI:** 10.3390/s23115167

**Published:** 2023-05-29

**Authors:** Degang Xu, Qing Song, Shiyu Fang, Yanrui Guo

**Affiliations:** School of Automation, Central South University, Changsha 410083, China

**Keywords:** 3D LiDAR point cloud, intelligent detection methods, normalization, shotcrete

## Abstract

In tunnel lining construction, the traditional manual wet spraying operation is labor-intensive and can be challenging to ensure consistent quality. To address this, this study proposes a LiDAR-based method for sensing the thickness of tunnel wet spray, which aims to improve efficiency and quality. The proposed method utilizes an adaptive point cloud standardization processing algorithm to address differing point cloud postures and missing data, and the segmented Lamé curve is employed to fit the tunnel design axis using the Gauss–Newton iteration method. This establishes a mathematical model of the tunnel section and enables the analysis and perception of the thickness of the tunnel to be wet sprayed through comparison with the actual inner contour line and the design line of the tunnel. Experimental results show that the proposed method is effective in sensing the thickness of tunnel wet spray, with important implications for promoting intelligent wet spraying operations, improving wet spraying quality, and reducing labor costs in tunnel lining construction.

## 1. Introduction

With the rapid development of 3D laser scanning technology, the application of laser technology is rapidly expanding and offering decreased costs and increased accuracy. This technology has various applications, including road detection [1,2], object recognition [3,4], surface reconstruction [5,6] and tunnel detection [7,8].

In China, the total length of highways is reported to be 4,846,500 km, including 17,236.1 km of highway tunnels as of 2018 [9]. It is estimated that by 2030, the total number of tunnels in operation in China will reach 17,000, exceeding 30,000 km in length [10]. Therefore, the development of intelligent tunnels in construction and monitoring is becoming increasingly urgent. The use of 3D laser scanning technology in tunnel construction and monitoring can improve construction efficiency, ensure safety, and reduce labor costs.

Laser technology has become an indispensable tool in the intelligent development of tunnels [11,12]. The use of laser scanners for acquiring 3D data of excavation surfaces in tunnels was first proposed by Lemy et al. [13]. They determined the displacement of the excavation surface by comparing the point clouds obtained at different times. This study demonstrated the potential of LiDAR in data collection during tunnel excavation. In subsequent years, Fekete et al. [7,14] used 3D laser scanning for quality control in drill and blast tunnels. LiDAR scanning allowed for precise monitoring of excavation and support installation during construction. More recent research has further explored the potential of LiDAR in tunnel construction activities, such as rock mass classification [15], drill bit wear detection [16], and automatic surveying of tunnel sections [17]. However, research on tunnel shotcrete is still lacking. Shotcrete is commonly used in the construction of railway and highway tunnels [18]. Tunnel shotcrete spraying is a process of rapidly applying shotcrete to the rock or concrete surface to prevent tunnel collapse during excavation [19,20]. Currently, shotcrete spraying is a manual process, resulting in unstable construction quality and slow construction progress. There is a need for further research on the intelligent construction of shotcrete in tunnels.

LiDAR-based 3D object detection is essential for automating tunnel shotcrete spraying, as it directly relates to understanding the tunnel environment. A previous study proposed a novel neural network structure based on LiDAR for detecting the area of tunnel shotcrete spraying [21], demonstrating the real-time monitoring of the tunnel profile and shotcrete area. Ranjbarnia [22] studied the effects of various parameters such as sprayed concrete thickness, soil geomechanical properties, tunnel depth, and fault plane dip angle using the 3D finite difference analysis algorithm and centrifuge physical model, but mainly focused on crossing faults in urban tunnels, which is not applicable for the construction analysis of tunnels in progress. Oreste [23] proposes a new calculation procedure based on the combined use of two calculation methods, convergence confinement and hyperstatic reaction methods, to analyze the factors of shotcrete and determine the trend of the lining safety factor over time. Unlike previous studies, our research focuses on detecting the thickness of shotcrete to enable intelligent detection for large spatial arch spraying processes in complex tunnel scenarios. To achieve this, we developed a LiDAR-based intelligent detection method. Our contribution lies in proposing a novel approach to address the specific challenge of shotcrete thickness detection.

In summary, the main contributions of this paper are as follows:

- This paper proposes a new method for the intelligent detection of wet shotcrete thickness on the tunnel arch surface during large spatial arch spraying processes in complex tunnel scenarios, which has not been previously explored. This method addresses the need for automated and accurate shotcrete spraying to improve the construction quality and progress.
- An innovative adaptive tunnel standardization processing algorithm is introduced, which mathematically describes the inner contour of the tunnel. This algorithm can accurately detect the tunnel arch surface, which is a prerequisite for detecting the thickness of shotcrete, and can adapt to different tunnel shapes and sizes.
- The proposed algorithm has demonstrated robust and reliable performance in detecting tunnel shotcrete thickness during tunnel construction in China. This method contributes significantly to the field of tunnel construction by improving construction quality and efficiency while reducing costs and risks associated with manual inspection.

The structure of this paper is as follows: Section 2 presents the problem and describes the process of data acquisition. In Section 3, an innovative adaptive method for normalizing point cloud data is proposed. Section 4 discusses a fitting method used to obtain a mathematical model of the inner contour of a large-scene tunnel. The experimental results are presented in Section 5. Finally, the paper is concluded with suggestions for further research in Section 6.

## 2. Material

### 2.1. Problem Description

In modern tunneling operations, it is essential to acquire detailed information about the shotcrete thickness of lining and installed support structures to ensure safety, long-term stability, and quality control. The lining construction [24], including initial and secondary lining, is an important tunnel supporting structure. The initial lining refers to shotcrete after laying steel arch support [25], which is widely used as a support element in underground building construction.

**Figure 1:**
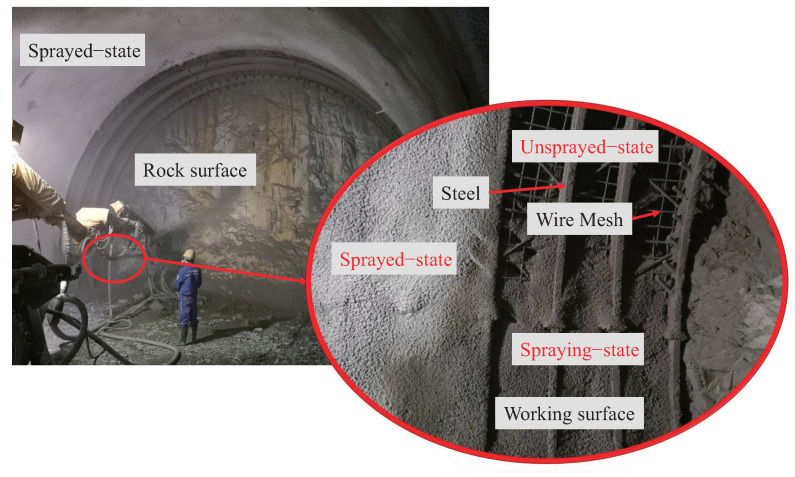
Current construction method and classification of wet shotcrete states. The red large circle indicates a magnification of the details in the small circle.

**Figure 2:**
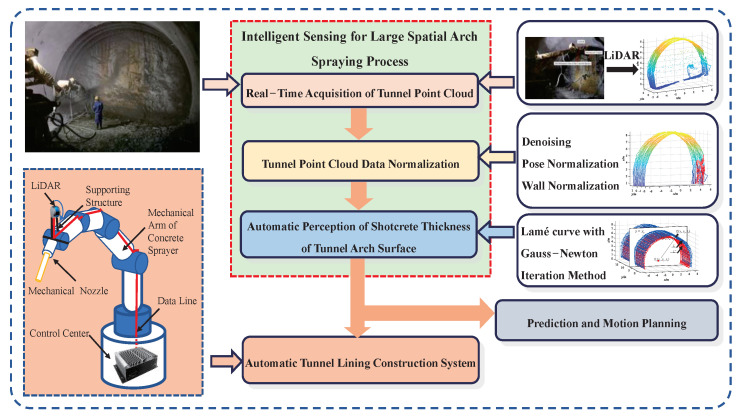
The solution of automatic tunnel lining construction system. The red box represents the components of "Intelligent Sensing for Large Spatial Arch Spraying Process", which include real-time point cloud acquisition, point cloud normalization, and intelligent perception for the steel arch. The arrows indicate the specific contents included in each component.

Tunnel shotcrete spraying is a process of spraying concrete at a high velocity onto the surface of a rock or steel structure in order to prevent tunnel collapse during excavation [26]. Currently, shotcrete spraying is a manual process, which involves an operator manually operating the mechanical arm for spraying, as shown in Figure 1. This method is highly dependent on the operator’s experience and skills, and the quality of the shotcrete largely depends on the operator. Furthermore, the manual operation exposes workers to a large amount of dust and heavy and dangerous work, which may cause health problems. Therefore, an automatic tunnel lining construction system, as shown in Figure 2, is necessary to automate the tunnel construction process.

In the automatic tunnel lining construction system, the wet shotcrete process of the tunnel arch is divided into three states: the unsprayed-state, the spraying-state, and the sprayed-state [27,28]. The unsprayed-state is the state in which the shotcrete has not yet been sprayed, the spraying-state is the state in which the shotcrete is being sprayed, and the sprayed-state is the state in which the shotcrete has been applied, as shown in Figure 1. LiDAR is used to collect tunnel point cloud data in these three states, which is then processed using denoising, correction, clustering, and compensation techniques to extract the tunnel arch points.

Afterward, a wet shotcrete thickness description model is established to determine the depth of shotcrete needed for the tunnel arch surface to be sprayed continuously. With the prediction and motion planning of the mechanical arm, an automatic tunnel lining construction system is proposed. This paper focuses on the intelligent sensing for the large spatial arch spraying process in complex tunnel scenarios, which lays the foundation for prediction and motion planning of the system.

### 2.2. Process of Data Acquirement

**Figure 3:**
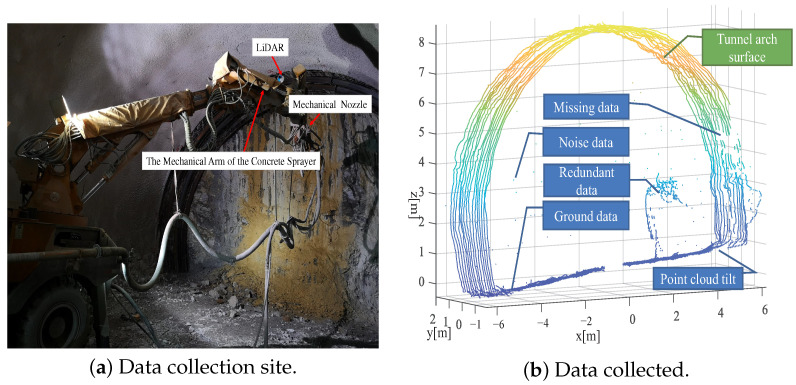
Data collection and analysis. (**a**) describes the data collection site. (**b**) displays a frame of tunnel point cloud data that includes various types of noise. The tunnel arch points are the target of what we want to extract.

The LiDAR used in this study is mounted on the mechanical arm of the concrete sprayer and integrates 16 laser/detector pairs in a compact housing for data collection [17]. In practice, the LiDAR is designed to be installed on the mechanical arm (as shown in Figure 3a). Since the shotcrete machine is generally oriented towards the working surface of the tunnel, during the wet spraying interval, when the mechanical arm stops moving, the LiDAR scans the tunnel to realize the measurement and perception of the environment. The original point cloud collected in reality is shown in Figure 3b, and there are mainly several issues with it:

(1) The point cloud is contaminated with noisy data due to severe dust pollution;
(2) The point cloud is tilted due to the movement of the mechanical arm and the installation of the LiDAR;
(3) The point cloud data contain redundant data;
(4) Some point cloud data are missing due to obstruction by obstacles.

To address the issues mentioned above with the collected tunnel point cloud data, an adaptive tunnel arch point extraction algorithm is proposed prior to sensing the shotcrete thickness of the lining.

## 3. Method

A point cloud is a vast collection of surface characteristic points of a target object that are directly obtained by LiDAR. However, due to the actual collection site conditions, noise interference is inevitable. Additionally, the presence of construction equipment, operators, rock waste, and other objects at the tunnel site may cause occlusion, which affects the quality of the point cloud to varying degrees. Moreover, the large volume of the tunnel point cloud and the existence of redundant information make it critical to extract the characteristics of the tunnel cross-section point cloud and reduce computational complexity. Therefore, standardizing the tunnel point cloud is crucial during the feature point extraction process to ensure accurate analysis and efficient computation.

### 3.1. Data Denoising

To filter out noise data caused by suspended concrete particles, we use a point cloud filtering algorithm based on the threshold neighborhood method proposed by Rusu and Cousins [29]. The algorithm selects neighboring points using a 3D Euclidean distance metric and terminates once a fixed number of neighbors $n_{nbrs}$ or all points within a bounding sphere of radius $r_{sph}$ have been found. The variables $n_{nbrs}$ and $r_{sph}$ control the size of the neighborhood selection. Subsequently, the mean μ and standard deviation σ of nearest neighbor distances are calculated, and points outside the μ±ϑ·σ range are removed, where the parameter ϑ adjusts the sensitivity of the threshold. In our implementation, we have found the optimal value of ϑ to be 1, and $n_{nbrs}=30$.

Furthermore, based on experiments with multiple datasets, we have shown that the μ±σ thresholds are effective in removing noise, where around 1% of the points are considered as noise.

### 3.2. Adaptive Point Cloud Pose Normalization

**Figure 4:**
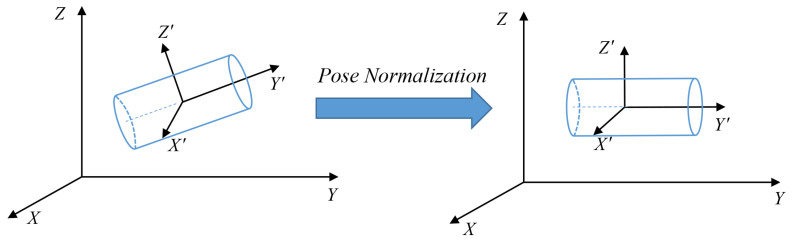
Schematic diagram of tunnel attitude standardization.

During the process of collecting radar data, the tunnel point cloud may be inclined to different degrees due to various installation reasons, as illustrated in the left schematic diagram in Figure 4. To enhance the stability and accuracy of tunnel wet spray state perception, it is crucial to transform the coordinates of the tunnel point cloud before the three-dimensional reconstruction of the tunnel. The pose normalization of the tunnel point cloud needs to be performed to adjust all the tunnel point clouds to the position shown on the right side of Figure 4. This process enables the alignment of the tunnel point cloud with the reference frame, facilitating a more precise three-dimensional reconstruction of the tunnel.

Considering the rigid body transformation characteristics of the tunnel, solving any part of the rotation transformation matrix can enable the complete rotation transformation of the tunnel point cloud. However, in the tunnel point cloud, the road point cloud exhibits significant planar characteristics, which can be utilized to correct the tunnel wall point cloud by solving the transformation matrix of the road point cloud. To extract the ground point cloud from the tunnel point cloud, we propose a planar extraction algorithm based on the M-estimator SAmple Consensus (MSAC) algorithm [30]. The MSAC algorithm optimizes the calculation method of the loss function of the RANSAC algorithm, addressing the RANSAC algorithm’s sensitivity to the threshold *T* selection of interior points. The loss function value of the RANSAC algorithm is represented by $C_{1}$. For the points inside the model, the loss function is 0, whereas for the points outside the model, a constant penalty is incurred. Thus, setting the threshold too high can result in poor estimation, while setting it too low can affect the robustness of the model.

(1) $$\begin{array}{c} C_{1}=\sum_{i}p_{1}\left(e_{i}^{2}\right) \end{array}$$

where *e* is the error function, and $p_{1}$ is the robust scale parameter, defined as

(2) $$p_{1}\left(e^{2}\right)=\left\{\begin{array}{cc} 0 & e^{2}<T^{2}\\ \mathrm{constant} & e^{2}\geq T^{2} \end{array}\right.$$

To address this issue, Torr and Zisserman [31] proposed a new loss function $C_{2}$ that can be minimized to obtain a more accurate model. The $C_{2}$ loss function is defined as

(3) $$\begin{array}{c} C_{2}=\sum_{i}p_{2}\left(e_{i}^{2}\right) \end{array}$$

where *e* is the error function, and $p_{2}$ is the robust standard error. The $p_{2}$ function assigns a weight to each data point based on its distance to the model, with larger weights assigned to points that are closer to the model. This effectively reduces the influence of outliers in the data and improves the accuracy of the model estimation.

(4) $$p_{2}\left(e^{2}\right)=\left\{\begin{array}{cc} e^{2}\; & e^{2}<T^{2}\\ T^{2} & e^{2}\geq T^{2} \end{array}\right.$$

**Figure 5:**
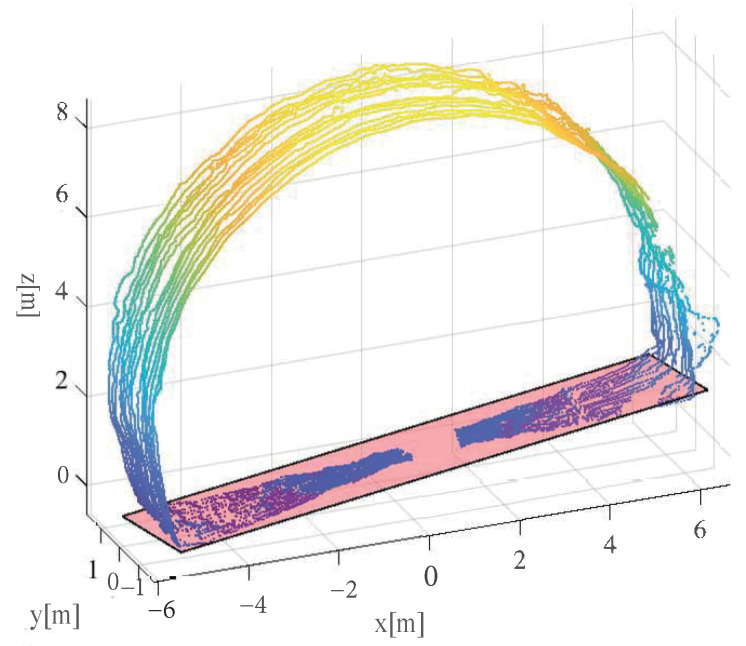
Fitting result of the MSAC algorithm for plane extraction. The pink plane represents the plane fitted by the algorithm.

To extract the plane of the point cloud data, the MSAC algorithm is utilized to obtain the plane equation, as shown in Equation (5). In this algorithm, a fixed penalty is given for out-of-model points, while for in-model points, the fitting effect of the model is considered to establish the most accurate model. Figure 5 shows the fitting result, which demonstrates that the extracted plane points correspond well with the road surface of the tunnel.

(5) $$a_{p}x+b_{p}y+c_{p}z+d_{p}=0$$

where $a_{p}$, $b_{p}$, $c_{p}$, and $d_{p}$ are the coefficients of the plane equation.

In Figure 5, the point cloud is observed to be inclined at a certain angle with respect to the X, Y, and Z axes. However, if the normal vector of the road point cloud is found to be parallel to the Z-axis, it indicates that this part of the point cloud has already undergone the attitude standardization process.

In the cases where the normal vector of the road point cloud is not parallel to the Z-axis, a point cloud correction algorithm based on continuous projection is proposed in this section.

The proposed point cloud correction algorithm based on continuous projection involves a series of steps. Firstly, the point cloud is projected onto the YOZ, XOY, and XOZ planes sequentially. Next, the α-shape algorithm [32] is employed to determine the boundary points of the point cloud on each plane, which are then used to identify the center line of the point cloud in the plane. The rotation angle of the point cloud in the plane is determined by the declination angle from the coordinate axis, and the transformation matrix is obtained using the Rodrigue formula [33]. Finally, the point cloud is corrected according to the transformation matrix.

**Figure 6:**
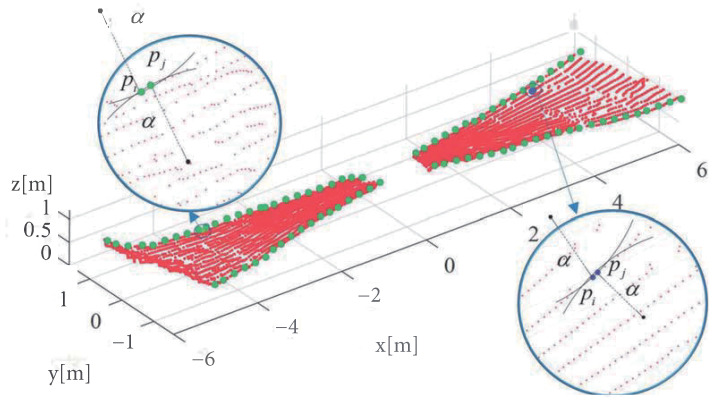
The alpha-shape algorithm criteria. The red points represent the point cloud data of the plane fitted by the MSAC algorithm in Figure 5, while the green points represent the boundary points detected by the α-shape algorithm. The points $p_{i}$ and $p_{j}$ on the left are the boundary points, whereas on the right, $p_{i}$ and $p_{j}$ are the internal points.

A common way to determine the boundary of a finite point set is through the α-shape algorithm [32]. For a finite point set, the algorithm forms a line segment between every two points and draws a circle with a diameter of the line segment. If one of the circles does not contain any other points except for the two points, then the two points are considered as two boundary points. The sum of these boundary points gives the boundary of the point cloud, as shown in Figure 6.

After obtaining the projected boundary points, a quadratic function is used to fit the boundary of the point cloud, as shown in Equation (6):

(6) $$\left[\begin{array}{c} y_{1}\\ y_{2}\\ \vdots \\ y_{n} \end{array}\right]=\left[\begin{array}{ccc} 1 & x_{1} & x_{1}^{2}\\ 1 & x_{2} & x_{2}^{2}\\ \vdots & \vdots & \vdots \\ 1 & x_{n} & x_{n}^{2} \end{array}\right]\left[\begin{array}{c} a_{q}\\ b_{q}\\ c_{q} \end{array}\right]+\left[\begin{array}{c} \epsilon_{1}\\ \epsilon_{2}\\ \vdots \\ \epsilon_{n} \end{array}\right]$$

where *n* is the number of boundary points; $x_{i}$ and $y_{i}$ are the coordinates of the *i*-th boundary point; $a_{q}$, $b_{q}$, and $c_{q}$ are the coefficients of the quadratic function; and the $\epsilon_{i}$ represent the residual error between the fitted curve and the original point cloud data.

**Figure 7:**
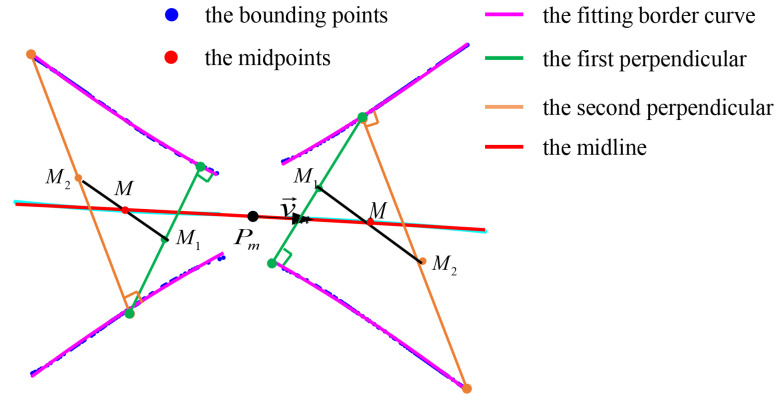
Determination of the midpoints on central axis. The green line represents one of the normal lines of the boundary. $M_{1}$ is the midpoint of the line segment between the intersection point of the normal line with the opposite boundary and the boundary itself. The orange line represents the normal line of the opposite boundary. $M_{2}$ is the midpoint of the line segment between the intersection point of the boundary’s normal line with the original boundary and the boundary itself. *M* is the intersection point of the line segments between $M_{1}$ and $M_{2}$, which represents the point on the midline of the plane, and the direction vector is $\vec{v}$.

To obtain the midline of the plane, we first use the uniform sampling method to obtain 50 sampling point sets on each boundary. For each sampling point, we record the normal line perpendicular to the boundary and the intersection point set with the opposite boundary. Then, we recalculate the intersection of the normal line at the intersection point on the boundary with the original boundary and record the intersection point set. Next, we take the midpoint of each line segment between the two intersection points and then calculate the midpoint of the two midpoints. The resulting set of midpoints is used to fit the midline using Equation (7). The entire process is illustrated in Figure 7.

(7) $$y=k_{m}x+b_{m}$$

where $k_{m}$ and $b_{m}$ are the parameters of a curve, and the RANSAC (RANdom SAmple Consensus) algorithm [34,35] is used to estimate the parameters of the model. Then, the inclination angle of the point cloud in the plane can be expressed by Equation (8).

(8) $$\theta =\arctan(k_{m})$$

Based on the Rodriguez rotation formula, the rotated vector ${\vec{v}}_{rot}$ of any vector ${\vec{v}}_{0}$ in space, rotated by an angle θ around a given rotation axis $\vec{n}$, can be expressed by Equation (9). This equation ensures the accuracy of the rotation operation.

(9) $${\vec{v}}_{rot}=M_{r}\left(\vec{n},\theta \right){\vec{v}}_{0}={\vec{v}}_{0}\cos\theta +\left(\vec{n}\times {\vec{v}}_{0}\right)\sin\theta +\left(1-\cos\theta \right)\left(\vec{n}\cdot {\vec{v}}_{0}\right)\vec{n}$$

where $M_{r}$ is the rotation matrix, which is defined as shown in Equation (10):

(10) $$M_{r}\left(\vec{n},\theta \right)=\left[\begin{array}{c} \;u^{2}\left(1-\cos\theta \right)+\cos\theta \;uv\left(1-\cos\theta \right)-w\sin\theta \;uw\left(1-\cos\theta \right)+v\sin\theta \\ uv\left(1-\cos\theta \right)+w\sin\theta \;\;\;v^{2}\left(1-\cos\theta \right)+\cos\theta \;\;uw\left(1-\cos\theta \right)-u\sin\theta \\ uw\left(1-\cos\theta \right)-v\sin\theta \;vw\left(1-\cos\theta \right)+u\sin\theta \;\;\;w^{2}\left(1-\cos\theta \right)+\cos\theta \end{array}\right]$$

**Figure 8:**
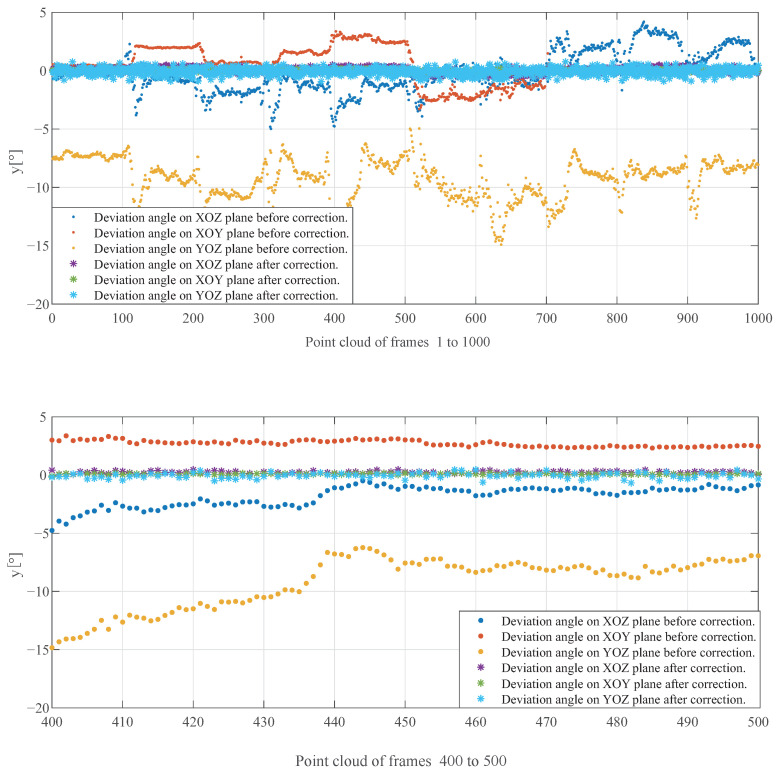
Comparison of deviation angle before and after correction. The x-axis represents different point cloud frames, while the y-axis represents the inclination angle.

To investigate the impact of the continuous projection algorithm proposed in this section on point cloud correction, this paper conducts an analysis of the inclination angles and coordinate axes of 1000 frames of tunnel point clouds with varying inclination degrees, before and after correction. The results are presented in Figure 8: “·” represents the tilt angle before correction, and “*” represents the tilt angle after correction. From the comparison of 1000 point clouds, it can be seen that there were varying degrees of tilt before correction, with the maximum deviation angle exceeding 15° in all directions. After point cloud correction, the point cloud was corrected well in all directions, with a tilt angle not exceeding 2°. The local enlargement images of 400 frames to 1000 frames further demonstrate the effectiveness of the algorithm correction.

### 3.3. Adaptive Point Cloud Wall Normalization

At the data collection site, construction equipment and workers are in continuous motion. Consequently, the point cloud collection process may result in some degree of obstruction of the tunnel wall, leading to data loss to varying extents. This can lead to subsequent issues in data processing and analysis. To address incomplete information, it is necessary to detect the area of the tunnel wall point cloud and interpolate missing data segments. This process is referred to as the wall standardization process of the tunnel point cloud in this paper.

In order to compensate for missing data in the tunnel wall, this paper presents an adaptive point cloud compensation algorithm based on an interpolation model. The algorithm consists of two main parts: automatic detection and automatic interpolation. During data collection, construction equipment and workers may obstruct the tunnel wall, leading to incomplete point cloud data. In order to address this issue, the proposed algorithm aims to automatically detect the missing areas of the tunnel wall point cloud and interpolate the missing data segments.

The adaptive point cloud compensation algorithm utilizes the first-order difference algorithm for the automatic detection of missing parts of the point cloud. Once the location of the missing point cloud is identified, the algorithm compensates for the missing segment through interpolation using the piecewise cubic Hermite interpolating polynomial (PCHIP) method, which is a type of piecewise polynomial interpolation that uses cubic Hermite polynomials to ensure the smoothness of the interpolated curve.

Assuming that $P_{k}{\left(x_{k},y_{k},z_{k}\right)}^{T}$ and $P_{k+1}{\left(x_{k+1},y_{k+1},z_{k+1}\right)}^{T}$ are two points of tunnel arch points after clustering, with a missing area between $P_{k}$ and $P_{k+1}$, the algorithm checks if $\left|z_{k}-z_{k+1}\right|\geq \delta$, where

(11) $$\;\begin{array}{c} \delta =\frac{\sum_{i=1}^{nums-1}\left|z_{i+1}-z_{i}\right|}{nums-1} \end{array}$$

Here, nums is the number of clusters of tunnel arch points, and δ is the threshold value for determining the missing area.

The cubic Hermite interpolation polynomial $H_{3}$ is required to satisfy Equation (12):

(12) $$\left\{\begin{array}{c} H_{3}\left(x_{k}\right)=\varphi \left(x_{k}\right),H_{3}\left(x_{k+1}\right)=\varphi \left(x_{k+1}\right)\\ {\dot{H}}_{3}\left(x_{k}\right)=\dot{\varphi }\left(x_{k}\right),{\dot{H}}_{3}\left(x_{k+1}\right)=\dot{\varphi }\left(x_{k+1}\right) \end{array}\right.$$

where φ(x) is the interpolation function, and $H_{3}\left(x\right)$ is the basis function of the piecewise cubic Hermite interpolation polynomial, which can be expressed as

(13) $$\;\begin{array}{c} \begin{array}{c} H_{3}\left(x\right)=\psi_{k}\left(x\right)\varphi \left(x_{k}\right)+\psi_{k+1}\left(x\right)\varphi \left(x_{k+1}\right)+\phi_{k}\left(x\right)\dot{\varphi }\left(x_{k}\right)+\phi_{k+1}\left(x\right)\dot{\varphi }\left(x_{k+1}\right) \end{array} \end{array}$$

where $\psi_{k}\left(x\right),\psi_{k+1}\left(x\right),\phi_{k}\left(x\right),\phi_{k+1}\left(x\right)$ is the cubic Hermitian interpolation basis function for nodes $x_{k}$ and $x_{k+1}$, and they and their derivative must satisfy Equation (14).

(14) $$\left[\begin{array}{c} \psi_{k}\left(x_{k}\right),\;\;\;\psi_{k}\left(x_{k+1}\right),\;\;{\dot{\psi }}_{k}\left(x_{k}\right),\;\;\;{\dot{\psi }}_{k}\left(x_{k+1}\right)\\ \psi_{k+1}\left(x_{k}\right),\psi_{k+1}\left(x_{k+1}\right),{\dot{\psi }}_{k+1}\left(x_{k}\right),{\dot{\psi }}_{k+1}\left(x_{k+1}\right)\\ \phi_{k}\left(x_{k}\right),\;\;\;\phi_{k}\left(x_{k+1}\right),\;\;\;\;{\dot{\phi }}_{k}\left(x_{k}\right),\;\;\;\;{\dot{\phi }}_{k}\left(x_{k+1}\right)\\ \phi_{k+1}\left(x_{k}\right),\;\;\phi_{k+1}\left(x_{k+1}\right),\;{\dot{\phi }}_{k+1}\left(x_{k}\right),\;\;{\dot{\phi }}_{k+1}\left(x_{k+1}\right) \end{array}\right]=I_{4}$$

where $I_{4}$ is a 4×4 identity matrix.

Under the constraints of Equation (14), $\psi_{k}\left(x\right)$ and $\phi_{k}\left(x\right)$ can be constructed as follows:

(15) $$\left\{\begin{array}{c} \psi_{k}\left(x\right)=\left(a_{\psi }x+b_{\psi }\right){\left(\frac{x-x_{k+1}}{x_{k}-x_{k+1}}\right)}^{2}\\ \phi_{k}\left(x\right)=a_{\phi }\left(x-x_{k}\right){\left(\frac{x-x_{k+1}}{x_{k}-x_{k+1}}\right)}^{2} \end{array}\right.$$

**Figure 9:**
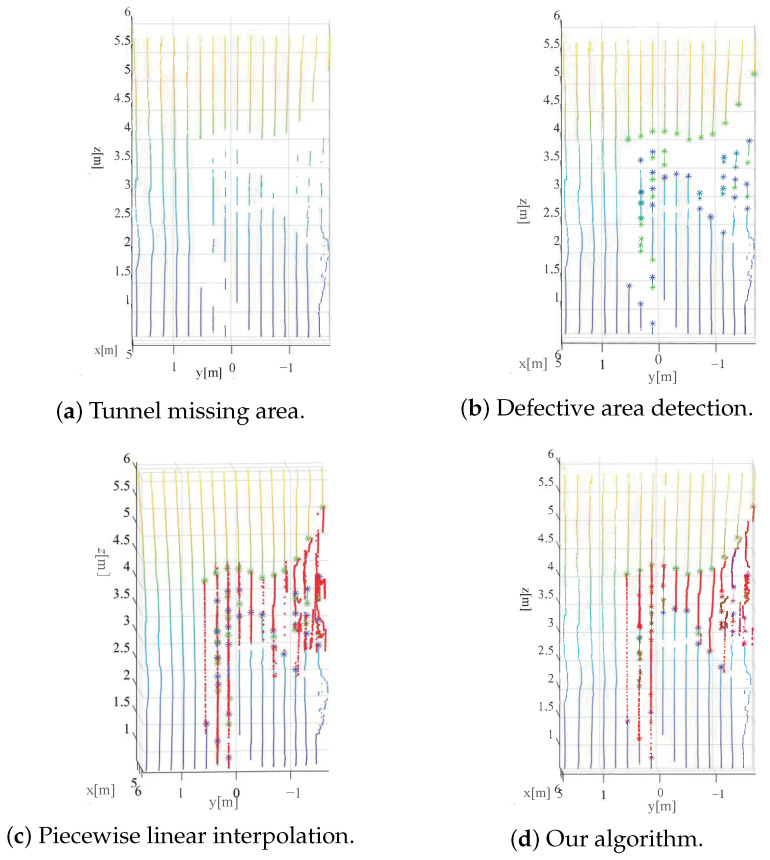
Compensation of missing parts, the red dots represent the interpolation points of the algorithm. (**a**) shows an area of data loss, where the tunnel wall point cloud is missing. (**b**) shows the markers for the missing parts of the tunnel in the point cloud; specifically, the green asterisk (*) point represents the starting point of the missing segment, while the blue asterisk (*) point represents the end point of the missing segment. (**c**) shows the result of piecewise linear interpolation. (**d**) displays the interpolation result of our proposed algorithm.

The parameters $a_{\psi }$, $b_{\psi }$, and $a_{\phi }$ can be obtained by using Equations (14) and (15) under the constraint of satisfying Equation (14). The expressions of $\psi_{k+1}\left(x\right)$ and $\phi_{k+1}\left(x\right)$ can be constructed in a similar way. This results in an interpolation polynomial between the endpoints $P_{k}$ and $P_{k+1}$, the interpolation results of which are shown in Figure 9. In Figure 9c,d, the red points represent the interpolated data points in the missing segments. It can be observed that the proposed algorithm not only preserves the general trend of the original data, but also achieves better data compensation accuracy with fewer wrongly interpolated data points, resulting in a superior data compensation effect.

## 4. Model

### 4.1. Theoretical Basis

#### 4.1.1. The Gauss–Newton Iteration Method

The cross-section of a tunnel is typically designed as an ellipse [36]. There are two types of algorithms to obtain an ellipse equation: non-iterative and iterative algorithms. Examples of non-iterative algorithms include the Lagrange multiplier-based method proposed in [37]. Iterative algorithms include the Gauss–Newton algorithm-based method introduced in [38,39]. However, due to the complex tunnel environment during the construction phase, the collected point cloud data are not suitable for non-iterative algorithms. Therefore, in this study, Taylor series expansion was used to approximate the nonlinear regression model and improve the regression coefficients by multiple iterations and corrections until the minimum residual sum of squares was achieved. This method is referred to as the Gauss–Newton iteration method.

It is assumed that Equation (16) represents a nonlinear regression model of an elliptical cross-section of a tunnel:

(16) $$\;\begin{array}{c} {\hat{y}}_{i}=f\left(x_{i},r\right)+\varepsilon_{i},\left(i=1,2,\cdots ,n\right) \end{array}$$

where $r={\left(r_{0},r_{1},\cdots ,r_{n_{r}}\right)}^{T}$ is an $n_{r}\times 1$ matrix of coefficients to be determined, and $\varepsilon_{i}$ represents the error term, which follows a normal distribution. The total number of points to be fitted is denoted by *n*, and $x_{i}$ is the *x*-coordinate of the *i*-th point, while ${\hat{y}}_{i}$ is the predicted value of $x_{i}$.

To obtain an initial value of the regression coefficient *r*, let $g^{\left(0\right)}={\left(g0^{\left(0\right)},g_{1}^{\left(0\right)},\cdots ,g_{n_{r}}^{\left(0\right)}\right)}^{T}$. Taylor expansion is used at $g^{\left(0\right)}$ in Equation (16), and the second order and above partial derivative terms are omitted to obtain Equation (17). This approach replaces the nonlinear regression model with a series expansion, and the regression coefficients of the nonlinear regression model are then iteratively updated and corrected until the minimum residual sum of squares is obtained using the Gauss–Newton iteration method.

(17) $$\;\begin{array}{c} f\left(x_{i},r\right)=f\left(x_{i},g^{\left(0\right)}\right)+{\sum_{j=0}^{n_{r}-1}\left[\frac{\partial f(x_{i},r)}{\partial r_{j}}\right]}_{r=g^{\left(0\right)}}\left(r_{j}-g_{j}^{\left(0\right)}\right) \end{array}$$

Equation (18) is obtained by combining Equations (16) and (17):

(18) $$\;\begin{array}{c} y_{i}^{\left(0\right)}\approx \sum_{j=0}^{n_{r}-1}G_{ij}^{\left(0\right)}b_{j}^{\left(0\right)}+\varepsilon_{i},\;\left(i=1,2,\cdots ,n\right) \end{array}$$

where

(19) $$\;\begin{array}{c} \left\{\begin{array}{c} y_{i}^{\left(0\right)}=y_{i}-f\left(x_{i},g^{\left(0\right)}\right)\\ G_{ij}^{\left(0\right)}={\left[\frac{\partial f(x_{i},r)}{\partial r_{j}}\right]}_{r=g^{\left(0\right)}}\\ b_{j}^{\left(0\right)}=\left(r_{j}-g_{j}^{\left(0\right)}\right) \end{array}\right. \end{array}$$

Equation (18) can be written in a more simplified matrix form as Equation (20):

(20) $$\;\begin{array}{c} Y^{\left(0\right)}\approx G^{\left(0\right)}b^{\left(0\right)}+\varepsilon \end{array}$$

where

(21) $$\;\begin{array}{c} \left\{\begin{array}{c} Y_{n\times 1}^{\left(0\right)}=\left[\begin{array}{c} y_{1}-f\left(x_{1},g^{\left(0\right)}\right)\\ \;\;\cdots \\ y_{n}-f\left(x_{n},g^{\left(0\right)}\right) \end{array}\right]\\ G_{n\times n_{r}}^{\left(0\right)}=\left[\begin{array}{c} G_{10}^{\left(0\right)}\;\cdots \;G_{1n_{r}-1}^{\left(0\right)}\\ \;\;\vdots \;\;\;\;\;\;\vdots \\ G_{n0}^{\left(0\right)}\;\cdots \;G_{nn_{r}-1}^{\left(0\right)} \end{array}\right]\\ b_{n_{r}\times 1}^{\left(0\right)}=\left[\begin{array}{c} b_{0}^{\left(0\right)}\\ \;\vdots \\ b_{n_{r}-1}^{\left(0\right)} \end{array}\right] \end{array}\right. \end{array}$$

Refine the correction coefficient $b^{\left(0\right)}$ using the least-squares method:

(22) $$\;\begin{array}{c} b^{\left(0\right)}={\left(G^{\left(0\right)T}G^{\left(0\right)}\right)}^{-1}G^{\left(0\right)T}Y^{\left(0\right)} \end{array}$$

The revised values for the regression coefficients $g^{\left(1\right)}$ can be obtained by using Equation (23):

(23) $$\;\begin{array}{c} g^{\left(1\right)}=g^{\left(0\right)}+b^{\left(0\right)} \end{array}$$

To update the correction coefficient $b^{\left(s\right)}$ at the *s*-th iteration, we can use the least-squares method, as shown in Equation (24). Then, the updated regression coefficients $g^{\left(s+1\right)}$ at the (s+1)-th iteration can be obtained by adding $b^{\left(s\right)}$ to $g^{\left(s\right)}$. This iterative process is repeated until the SSR (sum of squares of residual) is below a certain tolerable error *K*, which is given by Equation (25). More specifically, the iterative process continues until $\left|\frac{SSR^{\left(s\right)}-SSR^{\left(s-1\right)}}{SSR^{\left(s\right)}}\right|\leq K$, where $SSR^{\left(s\right)}$ and $SSR^{\left(s-1\right)}$ are the SSR values at the *s*-th and (s−1)-th iterations, respectively:

(24) $$\;\begin{array}{c} b^{\left(s\right)}={\left(G^{\left(s\right)T}G^{\left(s\right)}\right)}^{-1}G^{\left(s\right)T}Y^{\left(s\right)} \end{array}$$

(25) $$\;\begin{array}{c} SSR^{\left(s\right)}=\sum_{i=0}^{n}{\left[y_{i}-f\left(x_{i},g^{\left(s\right)}\right)\right]}^{2} \end{array}$$

where *s* represents the number of iterations.

#### 4.1.2. Analysis of Common Fitting Models

In China, the majority of large-scale tunnels are constructed as arched structures. Consequently, when addressing the problem of fitting the inner contour of a tunnel, arched models, such as the circle, ellipse, and Lamé curve, are commonly employed.

(1) Circle: A circle is the most fundamental geometric shape. For any circular figure with center $O_{c}\left(x_{c},y_{c}\right)$ and radius $R_{c}$, the standard equation of the circle is given by
  (26) $${\left(x-x_{c}\right)}^{2}+{\left(y-y_{c}\right)}^{2}=R_{c}^{2}$$
  Based on the findings reported in [40], circular structures are known to exhibit excellent pressure-bearing capacity. Therefore, tunnels excavated using shield machines commonly adopt circular structures.
(2) Ellipse: The actual tunnel environment is complex, and various factors such as geotechnical characteristics and surrounding rock mechanics need to be considered. Additionally, the deformation of the tunnel during use must be addressed. The elliptical structure can adjust its load capacity by changing the eccentricity and is commonly used in practical engineering. The equation for an ellipse is as follows:
  (27) $$\frac{{\left(x-x_{c}\right)}^{2}}{a^{2}}+\frac{{\left(y-y_{c}\right)}^{2}}{b^{2}}=1$$
  where $O_{c}\left(x_{c},y_{c}\right)$ is the center of the ellipse, *a* is the semimajor axis, and *b* is the semiminor axis.
  The load capacity of elliptical structural tunnels is closely related to the flatness of the ellipse, which can be described mathematically by the eccentricity *e*. In practical engineering, the eccentricity of the ellipse can be adjusted to change its load capacity. When *e* is closer to 0, the load capacity of the ellipse is stronger. Conversely, when *e* is closer to 1, the flatter the ellipse is, and the weaker its load capacity. This relationship between eccentricity and load capacity is important to consider when designing tunnels with elliptical cross-sections.
(3) The Lamé curve: This is also known as the hyperellipse [41], which is an extension of the ellipse. It has been widely used in tunnel engineering due to its adjustable shape parameters and excellent structural performance. The equation of the Lamé curve is given by
  (28) $${\left|\frac{x}{a}\right|}^{\eta }+{\left|\frac{y}{b}\right|}^{\eta }=1$$
  where *a* and *b* represent the major and minor axes of the Lamé curve, respectively, and η is the shape parameter that determines the shape of the curve.

**Figure 10:**
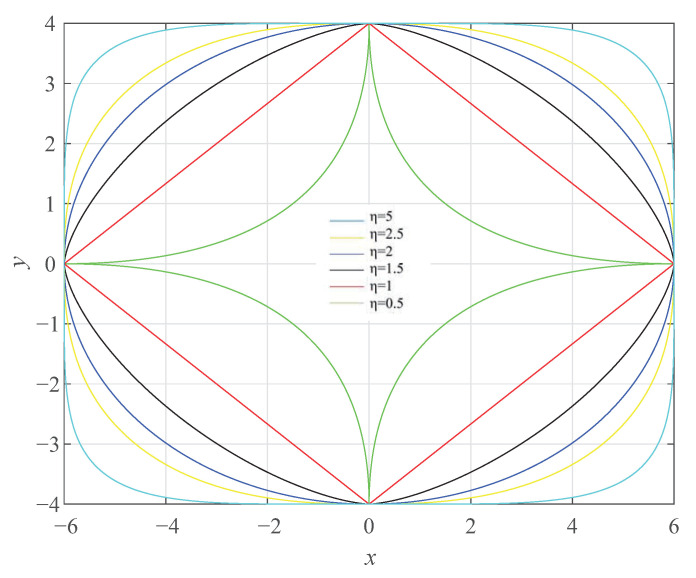
Lamé curve.

By adjusting the values of *a* and *b*, symmetric closed curves such as rectangles, circles, and hyperellipses can be obtained. When a=6.0 and b=4.0, hyperellipse curves of different orders can be obtained, as shown in Figure 10.

From Figure 10, it can be observed that when 0<η<1, the curve is concave inward and takes the shape of a four-pointed star. When η=1, the curve becomes a rhombus. For 1<η<2, the curve is convex, and the curvature increases as it approaches the vertices. When η=2, the curve becomes an ellipse, which is a circle if a=b. For η>2, the curve becomes a rectangle with rounded corners, and as η increases, it approaches a rectangle, which is also referred to as an ellipse in this case.

Based on the analysis of the circle, ellipse, and Lamé curve, it can be concluded that the Lamé curve is more suitable for fitting the inner contour of the tunnel section, depending on the specific shape of the tunnel. Therefore, this paper adopts the Lamé curve to fit the tunnel section.

### 4.2. Extraction of Cross-Section Point Cloud

The calculation of the central axis is a crucial step in obtaining the tunnel section, as it describes the direction of the tunnel, and each section is perpendicular to it. The central axis of the tunnel is typically calculated by projecting the point cloud of the inner wall of the tunnel. There are four commonly used methods to obtain the tunnel center line:

(1) Manual acquisition: low efficiency and large errors.
(2) Extracting the rails: not suitable for tunnels without steel rails.
(3) Calculating the tunnel boundary through data model fitting: limited by the tunnel shape.
(4) Fitting boundary lines on both sides of the tunnel: adopted in this paper due to the easy determination of boundary lines. The width is obtained by determining boundary lines, which are then shifted to center and averaged to obtain the center line of the tunnel.

In this study, the fourth method was chosen due to its simplicity and effectiveness, as the boundary lines on both sides of the tunnel can be easily determined. The method involves determining the width of the tunnel by finding the boundary lines on both sides of the point cloud and then shifting each boundary line towards the center. The center line of the tunnel is finally obtained by taking the average value of the shifted boundary lines.

### 4.3. Fitting of Cross-Section Point Cloud

Based on the above analysis, it is apparent that the Lamé curve model is more suitable for fitting the inner contour of the tunnel in wet spraying due to its robustness. However, fitting the entire tunnel outline without distinction and estimating the error can increase the amount of calculation and produce a large fitting error due to the different wet spray conditions of each area. To account for these conditions, a method for fitting the inner contour of the tunnel using segmented Lamé curves is proposed in this paper.

Initially, the sequential sampling method is used to take $n_{p}$ sampling points, and the Lamé curve is fitted to these points using the Gauss–Newton iteration method. The root mean square error (RMSE) of the fitting is then calculated, and if the threshold condition is met, the data are segmented, and sampling is performed from the left and right sides. The RMSE is recalculated until the threshold condition is exceeded, and the data segment fitted by the Lamé curve equation is obtained. Next, m sampling points are selected from the position where the data are interrupted, and this step is repeated until all points have been fitted.

(29) $$RMSE=\sqrt{\frac{1}{n_{p}}\sum_{i=1}^{i=n_{p}}{\left(observed_{i}-predicted_{i}\right)}^{2}}$$

**Figure 11:**
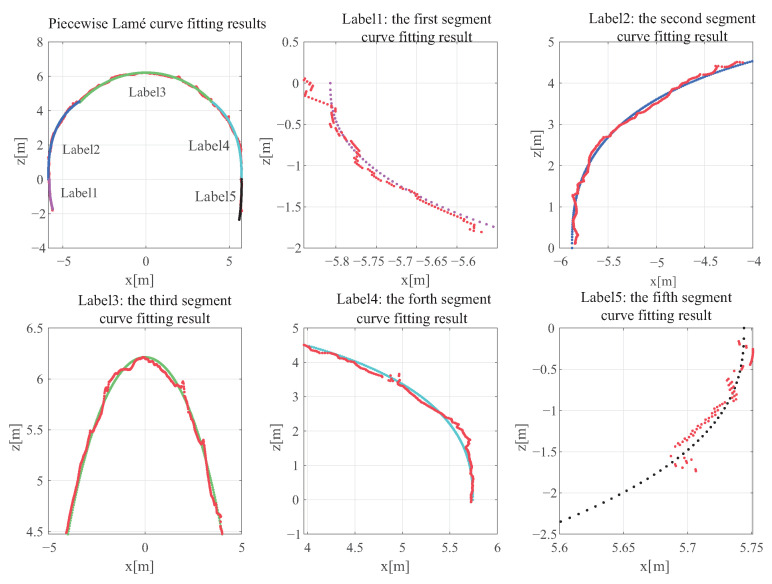
Piecewise Lamé curve fitting results. The labels 1 to 5 represent the fitting results of five curves. The red points indicate the original tunnel data point cloud, while the other color points represent the fitting curves of different regions.

For the inner contour curve of a specific section during the wet spraying process of the tunnel, the results of the fitting are presented in Figure 11.

In Figure 11, the red point cloud represents the actual inner contour curve of a section at a certain position in the tunnel, while the other lines of different colors represent different fitted Lamé curve segments. These curve segments are numbered from left to right, and Table 1 shows the fitting parameters and RMSE of these five curves.

After analyzing the fitting parameters in Table 1, it can be observed that the Lamé curve coefficients vary for different sections. However, the root mean square error of each section is within the ideal range, indicating that the model fitting effect is satisfactory.

### 4.4. Thickness Perception Model

**Figure 12:**
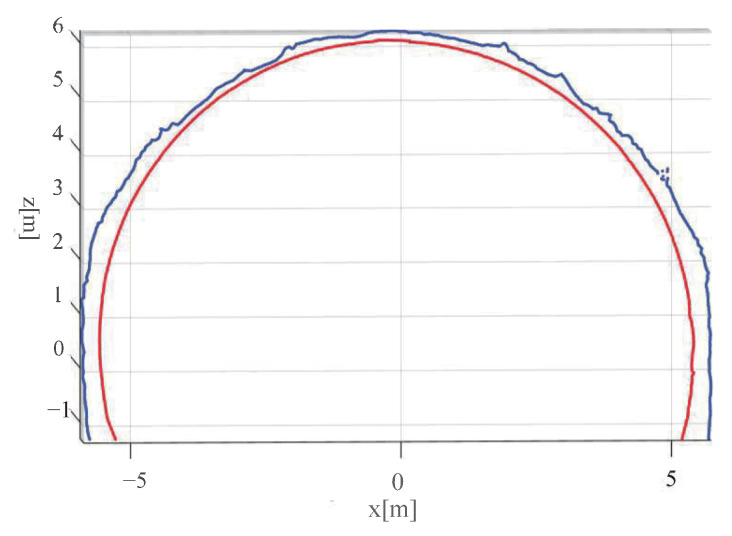
Front view of 3D wet shotcrete thickness description model. The blue curve denotes the cross-section of the tunnel to be wet sprayed, while the red curve denotes the tunnel lining design line.

**Figure 13:**
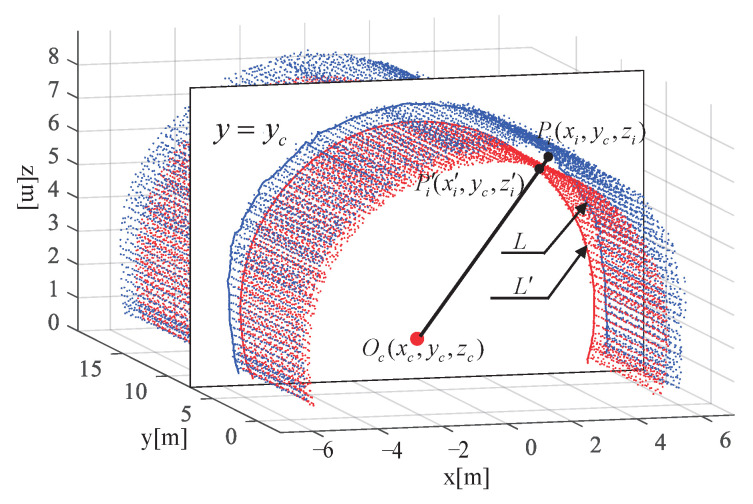
The 3D wet shotcrete thickness description model. The blue curve *L* represents the intersection curve of the unsprayed tunnel surface point cloud and the section, while the red curve $L^{\prime }$ represents the intersection curve of the designed surface and the section. Point $P_{i}$ is a point in *L*, while point $P_{i}^{\prime }$ is the corresponding mapping point of $P_{i}$ in $L^{\prime }$. The center of the tunnel at cross-section $y=y_{i}$ is denoted by $O_{c}$.

Figure 12 provides a front view of Figure 13, where the blue curve denotes the cross-section of the tunnel to be wet sprayed, while the red curve denotes the tunnel lining design line.

In Figure 13, the depth $d_{i}$ to be wet sprayed at a point $P_{i}$ on the tunnel section can be observed. The blue curve *L* represents the outline of the tunnel section, while the red curve $L^{\prime }$ represents the inner outline of the tunnel lining design.

$O_{c}$ denotes the central point of the section structure, while $P_{i}^{\prime }$ is the intersection point between $\vec{O_{c}P_{i}}$ and the inner contour line of the tunnel lining design. The thickness to be wet sprayed at point $P_{i}^{\prime }$ is then calculated as $d_{i}=∥P_{i}{P_{i}}^{\prime }∥$.

During the fitting of the tunnel contour using the segmental Lamé curve, each point $P_{i}$ on the contour was mapped to a point $P_{i}^{\prime }$ on the designed inner contour line of the tunnel. Therefore, $P_{i}^{\prime }$ is a point on the Lamé curve and satisfies the equation of the Lamé curve for its corresponding segment. From the 3D wet shotcrete thickness description model, we have that the thickness to be wet sprayed at point $P_{i}^{\prime }$ is given by $d_{i}=∥P_{i}P_{i}^{\prime }∥$. Hence, $P_{i}^{\prime }$ satisfies both the equation of the segmental Lamé curve and the equation of the distance between $P_{i}$ and $P_{i}^{\prime }$. Therefore, we can write the two equations in a system of equations as follows:

(30) $$\;\begin{array}{c} \left\{\begin{array}{cc} \; & {\left|\frac{x-x_{c}}{a}\right|}^{\eta }+{\left|\frac{z-z_{c}}{b}\right|}^{\eta }=1\\ \; & \vec{O_{c}P_{i}}=\lambda \vec{O_{c}{P^{\prime }}_{i}} \end{array}\right. \end{array}$$

where λ represents an arbitrary constant.

## 5. Experiment

**Figure 14:**
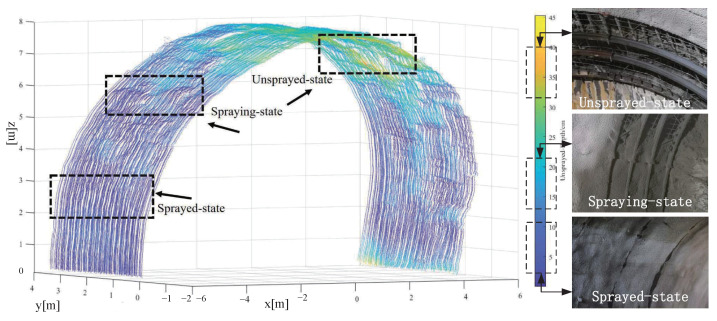
Experimental results.

The proposed algorithm has been implemented in a highway tunnel construction project in China, and the experimental results are presented in Figure 14.

As previously described, there are three stages of shotcreting, each requiring a different depth of concrete to be sprayed onto the tunnel surface. These depths are shown in Figure 14 for each respective stage. For the tunnel area in its three states of undried spraying, wet spraying, and dried spraying, we sampled a typical area of 1 m × 3 m × 0.2 m from the experimental results for verification and analysis.

(1) For the sampled areas in the unsprayed-state, which included 15,973 points, the average depth to be sprayed was 39.85 cm, which is close to the maximum design thickness of 40 cm for the concrete. Due to the varying depth of rock excavation in the unsprayed area, the depth to be sprayed for each point differed, and there was no specific pattern to follow. This reflects the actual construction conditions in industrial settings.
(2) In the sampled areas of the sprayed-state, which included 17,345 points, the average depth to be sprayed was 15.48 cm, with a maximum depth of 23.95 cm. This increase in depth from the bottom up is consistent with the wet spraying process, where spraying is done in a bottom-up sequence, and reflects the actual construction rules.
(3) For the sampled areas in the sprayed-state, which included 17,189 points, the average depth to be sprayed was 3.51 cm, with a maximum depth of 4.83 cm. In total, 90.43% of the sampling points were concentrated within the range of 3.5 ± 0.5 cm, and only 1.37% of the sampling points exceeded 4.5 cm, which is consistent with the on-site working conditions.

The consistency between the unsprayed depth of different states and the actual construction site indicates the reliability of the proposed algorithm.

**Figure 15:**
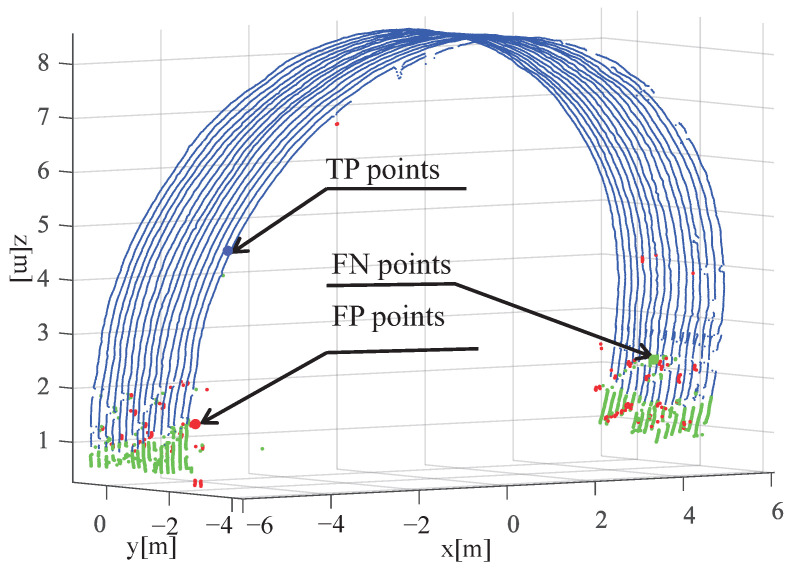
Different types of points in a certain section of the tunnel. The TP points and FP points represent tunnel surface points that were labeled correctly and incorrectly, respectively. The FN points were the points that were falsely labeled as non-tunnel surface points.

To evaluate the accuracy of our model, the tunnel arch points extracted in the previous step were manually labeled, as shown in Figure 15.

In Figure 15, the variables TP (True-Positive) marked in blue and FP (False-Positive) marked in red indicate the number of points that were labeled correctly and incorrectly as tunnel surface points, respectively. The variable FN (False-Negative) marked in green represents the number of points that were falsely labeled as non-tunnel surface points. To evaluate the performance of the model, the precision, recall, and F-score criteria used by Yang et al. [42,43] are adopted.

(31) $$\;\begin{array}{c} \left\{\begin{array}{c} precision=\frac{TP}{TP+FP},\\ recall=\frac{TP}{TP+FN},\\ F-score=2\cdot \frac{precision\cdot recall}{precision+recall}. \end{array}\right. \end{array}$$

Table 2 presents the performance evaluation of the proposed algorithm for tunnel surface extraction and compensation using the criteria mentioned above, including precision, recall, and F-score.

Furthermore, we compared the average precision, recall, and F-score rates of the results obtained by different methods and obtained intuitive comparison results, as shown in Table 3. Based on the experimental results, the proposed method demonstrated higher precision and recall rates than the control group.

The region-growing method [17] is applied to segment the rock surface from the tunnel point cloud. A curvature threshold is used with the region-growing algorithm to extract the rock surface of the tunnel. Additionally, the height threshold is used after DBSCAN to remove the miscellaneous points on the left and right walls of the tunnel. This method achieves an average precision, recall, and F-score of 80.7%, 79.5%, and 80.1%, respectively.

The elliptical cylinder model algorithm [36] differs from region growth algorithms in that it uses the central axis of the fit to divide the region into two parts. Subsequently, the elliptical fitting surface of the tunnel region is obtained through iteration to achieve the filtering of inner wall non-points. This method achieves an average precision, recall, and F-score of 83.7%, 82.9%, and 83.3%, respectively.

The continuous central axis is extracted by 2D projection [44], and then an interpolation algorithm based on quadric parametric surface fitting, using the BaySAC (Bayesian SAmpling Consensus) algorithm, is proposed to compute the cross-sectional point when it cannot be acquired directly from the tunnel points along the extraction direction of interest. This method achieves an average precision, recall, and F-score of 79.2%, 80.1%, and 79.6%, respectively.

By analyzing six sampled areas under different wet spraying conditions, our experimental results indicate that the algorithm achieved an average precision, recall, and F-score of 93.6%, 91.6%, and 92.6%, respectively. This demonstrates that our approach is better suited for analyzing complex wet spraying tunnel walls.

**Figure 16:**
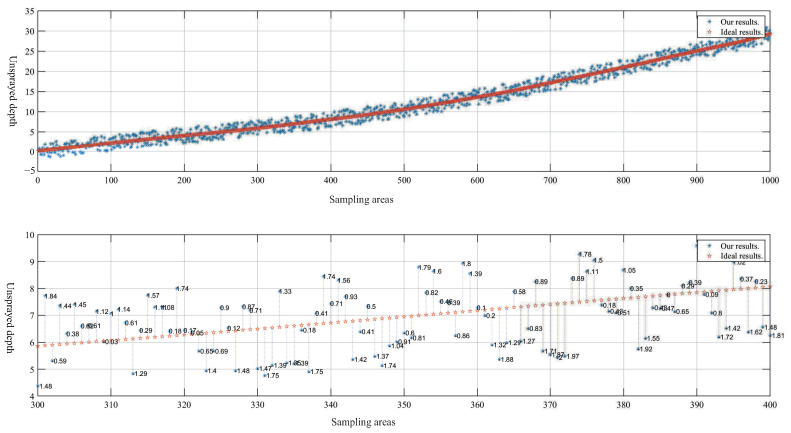
Comparison of the ideal unsprayed depth and the results obtained by our algorithm. The x-axis represents the sampling area, the y-axis represents the unsprayed depth, and the point marked with a star (✩) represents the ideal unsprayed depth. The point marked with an asterisk (*) represents the result obtained by our algorithm, and the number next to the point represents the absolute error between the unsprayed depth obtained by the proposed algorithm and the ideal unsprayed depth.

To verify the accuracy of the proposed method in determining the depth of concrete needed to be sprayed on the tunnel surface, a series of experiments was conducted in a real tunnel. The theoretical unsprayed depth ${D_{i}}^{\prime }$ obtained from engineering design was compared to the algorithmic results $D_{i}$ as shown in Figure 16.

The absolute average error MAD of the 1000 sampled areas is 0.989 cm, which demonstrates that the proposed algorithm meets the accuracy requirements of engineering design and confirms its reliability.

(32) $$\;\begin{array}{c} MAD=\frac{1}{n}\sum_{i=1}^{n}|{D_{i}}^{\prime }-D_{i}| \end{array}$$

## 6. Conclusions

In this paper, we proposed an algorithm for analyzing the area of interest in a tunnel point cloud. We used a continuous projection point cloud correction algorithm to process the attitude of the inclined point cloud during acquisition and developed an adaptive point cloud compensation algorithm to overcome data loss caused by occlusion. Due to the irregular cross-sections of the large tunnel scene and large space, we proposed fitting the segment Lamé curve to mathematically describe the inner contour line of the tunnel, instead of using a simple elliptical cylinder or cylinder model. We then compared the tunnel design line to analyze the thickness of the tunnel to be wet sprayed, allowing for accurate assessment of the tunnel construction. The proposed algorithm has been shown to effectively evaluate the depth of concrete required to be sprayed on the tunnel surface, with an absolute average error of 0.989cm, meeting the precise requirements of engineering design and demonstrating its reliability.

We plan to continue to conduct research on tunnel wet spraying processes, specifically analyzing different scenarios and studying the more complex construction process of long bend tunnels. Our aim is to establish a comprehensive system for monitoring the depth of wet spraying in tunnels, as well as to investigate the positioning issue of mobile LiDAR data to achieve higher real-time detection algorithms.

## Figures and Tables

**Table 1:** Piecewise Lamé curve fitting parameters.

ID	1	2	3	4	5
***a***	5.81	5.87	5.75	5.74	5.74
***b***	4.67	5.37	6.21	5.54	7.05
** η **	2.50	2.65	2.00	2.48	2.53
** RMSE **	0.0264	0.0476	0.0564	0.0570	0.0425

**Table 2:** Assessment of the depth of concrete required to be sprayed on the tunnel surface in six sample areas.

ID	1	2	3	4	5	6	Average
Precision	0.918	0.924	0.987	0.933	0.941	0.912	0.936
Recall	0.906	0.915	0.934	0.920	0.903	0.917	0.916
F-score	0.912	0.920	0.960	0.927	0.922	0.915	0.926

**Table 3:** Comparison of precision and recall rate of each method.

Method	Precision	Recall	F-Score
Region-growing	0.807	0.795	0.801
Elliptic cylindrical model	0.837	0.829	0.833
2D projection + BaySAC	0.792	0.801	0.796
Our method	0.936	0.916	0.926

## Data Availability

Data sharing not applicable.
